# Reconstruction of penile skin defect using negative pressure therapy, spiraling full-thickness skin grafts, and nanofat grafting: a case report

**DOI:** 10.1093/jscr/rjae663

**Published:** 2024-11-26

**Authors:** Diana Marcela Cadena Buitrago, Manuel Felipe Aljure Díaz, Ana María Camargo López, Juan Sebastián Afanador Ardila, Jorge Luis Corcho Acosta

**Affiliations:** Plastic and Reconstructive Surgery Department, Hospital Universitario La Samaritana, Bogotá 110311, Colombia; Plastic and Reconstructive Surgery Department, Hospital Universitario La Samaritana, Bogotá 110311, Colombia; Universidad el Bosque, Bogotá, Colombia; Plastic and Reconstructive Surgery Department, Hospital Universitario La Samaritana, Bogotá 110311, Colombia; Universidad el Bosque, Bogotá, Colombia; Plastic and Reconstructive Surgery Department, Hospital Universitario La Samaritana, Bogotá 110311, Colombia; Universidad el Bosque, Bogotá, Colombia; Plastic and Reconstructive Surgery Department, Hospital Universitario La Samaritana, Bogotá 110311, Colombia; Universidad del Rosario, Bogotá, Colombia

**Keywords:** fat grafts, skin graftings, negative pressure wound therapy, penile skin reconstruction

## Abstract

The penis is a structure that requires both anatomical and functional reconstruction. Being a three-dimensional structure that changes in volume, it presents a reconstructive challenge for the plastic surgeon. Currently, various alternatives are available for covering these complex defects, such as grafts, flaps, and dermal matrices. The objective of this study is to present a case report describing a complex post-traumatic penile defect in which three combined strategies were implemented for the first time (spiral full-thickness grafts, nanolipoinjection, and negative pressure therapy). These strategies have been described in the literature for defect coverage, achieving satisfactory anatomical and functional results.

## Introduction

The penis requires reconstruction for coverage defects resulting from trauma, burns, necrotizing infections, or paraffinomas. Its three-dimensional structure changes with volume fluctuations and contains multiple delicate components and poses a significant reconstructive challenge for plastic surgeons [1].

The possibilities for reconstruction with local or regional flaps for coverage defects that maintain both function and an adequate appearance are limited [1, 2]. Currently, there are various alternatives for covering these defects, including partial-thickness skin grafts (PTSG), full-thickness skin grafts (FTSG), and dermal matrices [3]. Since the penis changes size during erection, FTSG are preferred, not only for their greater elasticity and resistance to trauma or friction but also for their lower secondary contraction [4, 5]. Although FTSGs are preferred, it is important to optimize the recipient bed to offer a greater possibility of graft survival and integration. For this reason, different strategies have been implemented, mainly the negative pressure therapy [6–8].

The objective of this study is to describe the clinical case of a young patient who presented a circumferential coverage defect due to avulsion of the penile skin, which was reconstructed using a spiral full-thickness graft, nanofat grafting, and negative pressure therapy.

## Clinical case

A 26-year-old male with no significant medical history suffered severe polytrauma from a motorcycle accident, including abdominopelvic trauma with coverage defects in the lower abdomen, pelvis, scrotum, and thighs. After surgery control damage, he was then transferred to our institution for further evaluation and management in the ICU. From day one of the polytrauma, the patient received broad-spectrum empirical antibiotic coverage with piperacillin-tazobactam, which was subsequently de-escalated according to the patient’s clinical course by the infectious diseases service.

**Figure 1:**
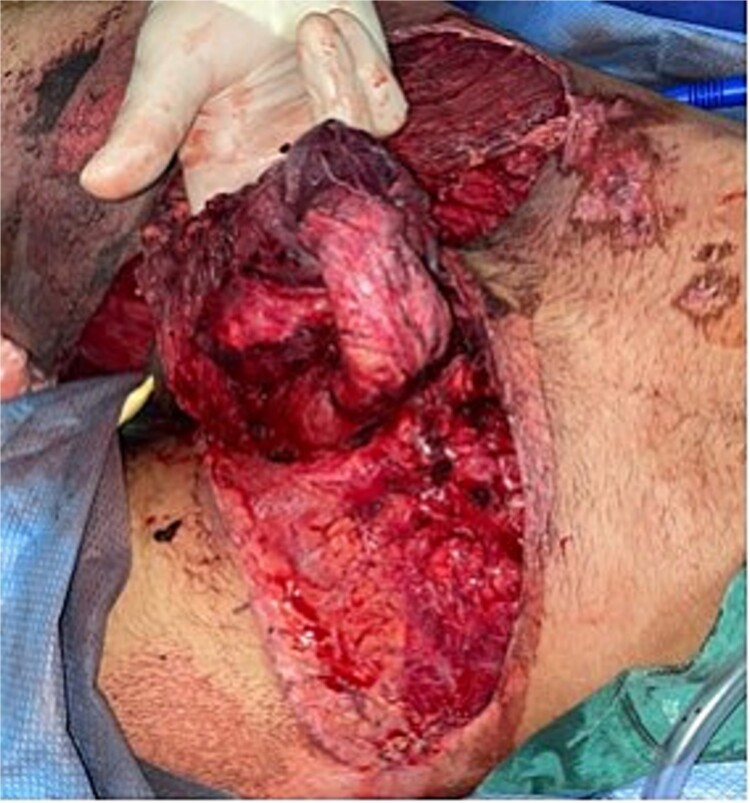
Complete scrotal exposure, denuded penis without exposure of the vascular pedicle at the frenulum, complete exposure of the base of the corpora cavernosa without involvement of the albuginea.

**Figure 2:**
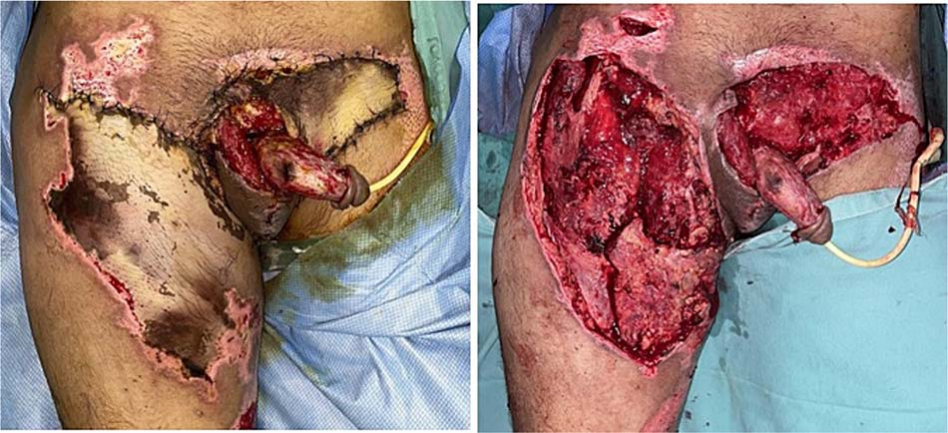
Necrosis and escharectomy of fasciocutaneous flaps on the right thigh, left inguinal region, and suprapubic area.

**Figure 3:**
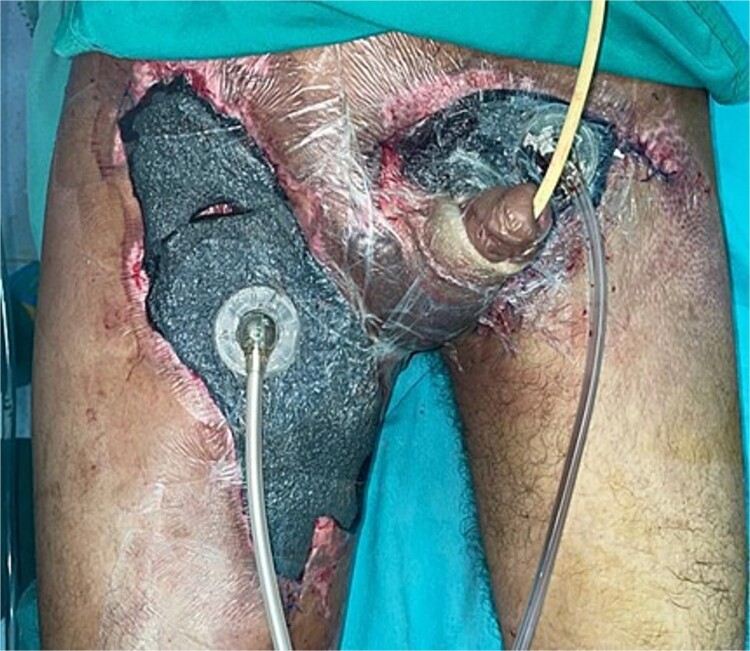
Application of circumferential negative pressure therapy with WhiteFoam to the circular defect at the penile body and granufoam to the right thigh, left inguinal region, and suprapubic area.

The Institutional initial intraoperative evaluation revealed complete right scrotal exposure, a denuded penis without vascular pedicle exposure, and complete exposure of the corpora cavernosa bases. The right testicle was fixed to the scrotal sac, while the left side showed an absence of the spermatic cord and testicle. Plastic surgery noted avulsion flaps in the left inguinal region, muscle exposure with necrotic Gracilis and Sartorius muscles, and avulsion flaps of penile and scrotal skin with ischemia (Fig. 1). Local fasciocutaneous flaps were initially advanced, but 90% of the scrotum necrosed, necessitating further debridement and scrotal skin flap advancement. Two weeks later, necrosis of additional fasciocutaneous flaps led to escharectomy and the application of negative pressure wound therapy (Figs 2 and 3). No cultures of the necrotic tissue were taken. To promote granulation tissue formation, three negative pressure wound therapy changes were performed prior to definitive reconstruction, during the final change, microbiological cultures were obtained from the defect bed at the penile and inguinal regions, showing no evidence of active infection. Consequently, the patient underwent definitive reconstruction 30 days after the initial trauma and 21 days after the escharectomy.

During the reconstructive surgery, it was possible to observe, after removing the negative pressure wound therapy, a full-thickness defect in the penis with a circumferential raw area involving 90% of the penile body, with exposed delicate structures.

**Figure 4:**
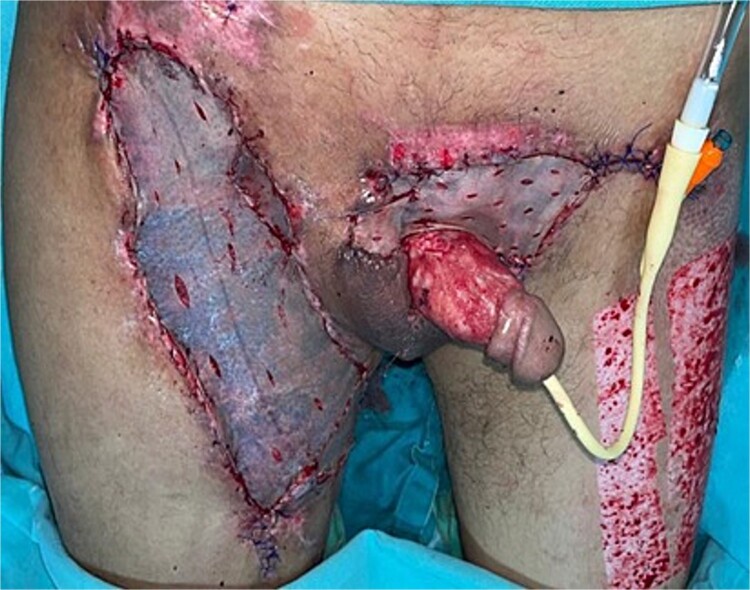
Removal of negative pressure therapy and placement of partial-thickness grafts in the raw areas of the right thigh, left inguinal region, and suprapubic area.

**Figure 5:**
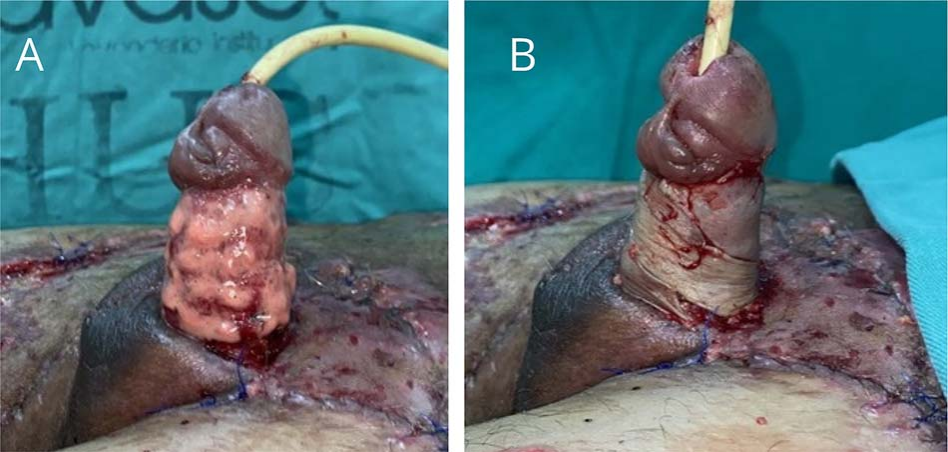
(A) Application of nanofat grafts circumferentially around the penile body. (B) Placement of spiral full-thickness grafts to cover the circumferential defect of the penile body.

Reconstruction required thin, elastic coverage that could resist trauma and friction, adapt to size changes, and provide stability and sensitivity. To achieve this, a combined technique was used, including spiral full-thickness grafts, nanofat grafting, and negative pressure therapy, as described below:

The defect was irrigated and debrided with 1000 ml of 0.9% saline solution, artificial erection was induced by urology, conventional liposuction was performed on the outer flanks and thighs, and the fat was decanted and filtered to produce nanofat grafts. These were applied superficially using an 18 FR needle. Two full-thickness grafts, each measuring 10 cm in length and 3 cm in width, were harvested from the inner left thigh and arranged in a spiral fashion according to the technique described by Thaddeus *et al.* [9]. They were then fixed with absorbable monofilament sutures. Finally, an interface with impregnated gauze with nitrofurazone and a negative pressure system was applied. Specifically, on the penis, a WhiteFoam (polyvinyl alcohol) foam dressing is applied over the full-thickness grafts to keep them in position. The dressing is positioned circumferentially around the shaft of the penis and secured with the transparent film of the negative pressure wound therapy system at −75 mmHg with continuous therapy (Figs 4 and 5).

**Figure 6:**
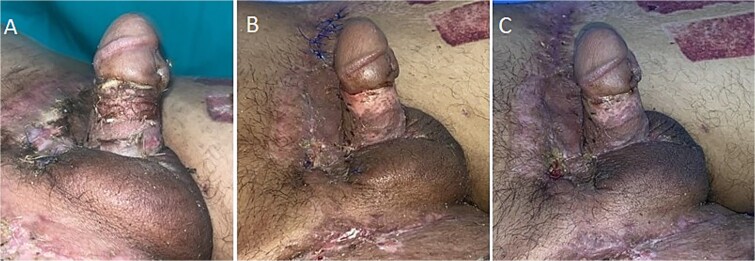
Postoperative images on days 7 (A), 14 (B), and 21 (C) following reconstruction.

One week later, the grafts were uncovered, showing more than 95% integration, without complications. Occlusive dressing with impregnated gauze was applied, the urinary catheter was removed, and once the patient achieved spontaneous voiding, he was discharged. Before reconstruction, the erect penis measured approximately 12 cm in length, and after surgery, a length of 2 cm less was achieved during the follow-up period (Fig. 6).

## Discussion

The reconstructive challenge in circumferential defects of the penis is to ensure a surface resistant to trauma and friction, with flexibility and extensibility [10]. Although various articles in the literature describe different surgical techniques, no consensus currently exists for determining a management algorithm for these types of defects. However, different approaches have been described.

One reconstructive strategy is partial-thickness grafts, as described by multiple authors who highlight the main advantages of replacing tissue with similar characteristics and the availability of multiple donor sites [3–8]. However, this reconstruction method does not provide sufficient coverage for a moist and friction-prone area, and the impact of secondary contraction on the natural shape and contour of the penis and/or the occurrence of postoperative retractions could not be determined. In our patient, we opted use spiral full-thickness grafts as they have shown satisfactory results, as their disposition allows for penile elongation during erection [9].

It is also important to induce artificial erection before placement to achieve the maximum possible dimension and thus establish the true defect requiring coverage. Multiple methods have been described for this, including the administration of Alprostadil and Tadalafil, allowing for adequate correlation between penile and graft size [10–12]. In our patient, the urology team performed intracavernous injections of 0.9% saline solution, achieving partial erection and allowing the actual size of the defect to be established.

Due to the potential problems mentioned by Thaddeus *et al.* [9], negative pressure therapy was used preoperatively and postoperatively. Its advantages include stimulating angiogenesis and regulating moisture and temperature in the graft, improving its integration. In our patient, no complications associated with this therapy were observed, and it facilitated graft stabilization by immobilizing it and optimizing the environment for healing.

Finally, although the literature does not recommend routine use of nanofat, its application on large, irregular surfaces like the one presented in this case can promote the formation of new vessels and fibroblasts [13]. In our patient, this strategy was implemented to accelerate the integration process, obtaining favorable results with more than 95% integration one week after the surgical procedure.

## Conclusion

Reconstructing penile defects poses significant challenges due to the lack of standardized protocols. Several surgical approaches, such as partial or full-thickness grafts, dermal matrices, and flaps have been proposed. This case report highlights a surgical technique that achieved successful anatomical and functional restoration, providing an elastic, extensible surface capable of accommodating changes in penile size. The findings suggest that a circumferential full-thickness graft, in combination with nanofat and negative pressure therapy, could be a promising reconstructive alternative.

## References

[1] Fuller TW , Theisen KM, Shah A, et al. Surgical man-agement of an adult’s acquired buried penis. Curr Urol Rep 2018;19:2–7. 10.1007/s11934-018-0768-1. 29492732

[2] Garaffa G , Christopher N, Ralph DJ. The management of genital lymphoedema. BJUI International 2007;102:480–4. 10.1111/j.1464-410X.2008.07559.x. 18325055

[3] Triana Junco P , Dore M, Nuñez Cerezo V, et al. Penile reconstruction with skin grafts and dermal matrices: indications and management. European J Pediatr Surg Rep 2017;5:e47–50. 10.1055/s-0037-1606282. PMC5578817 28868232

[4] Thakar HJ , Dugi DD III. Skin grafting of the penis. Urologic Clinics of North American 2013;40:439–48. 10.1016/j.ucl.2013.04.004. 23905942

[5] White N , Hettiaratchy S, Papini RP. The choice of split-thickness skin graft donor site: patients’ and surgeons’ preferences. Plast Reconstr Surg 2003;112:933–4. 10.1097/01.PRS.0000074491.30012.CE. 12960897

[6] Weinfeld AB , Kelley P, Yuksel E, et al. Circumferential negative-pressure dressing (VAC) to bolster skin grafts in the reconstruction of the penile shaft and scrotum. Ann Plast Surg 2005;54:178–83. 10.1097/01.sap.0000143606.39693.3f. 15655470

[7] Zhao JC , Xian CJ, Yu JA, et al. Reconstruction of infected and denuded scrotum and penis by combined application of negative pressure wound therapy and split-thickness skin grafting. Int Wound J 2013;10:407–10. 10.1111/j.1742-481X.2012.00997.x. 22672131 PMC7950686

[8] Stokes TH , Follmar KE, Silverstein AD, et al. Use of negative-pressure dressings and split-thickness skin grafts following penile shaft reduction and reduction scrotoplasty in the management of penoscrotal elephantiasis. Ann Plast Surg 2006;56:649–53. 10.1097/01.sap.0000202826.61782.c9. 16721079

[9] Prasetyono TOH . One-sheet spiraling full thickness skin graft for penile resurfacing after paraffinoma excision. Medical Journal of Indonesia 2011;20:222–5. 10.13181/mji.v20i3.450.

[10] Iblher N , Fritsche HM, Katzenwadel A, et al. Refinements in reconstruction of penile skin loss using intra-operative prostaglandin injections, postoperative tadalafil application and negative pressure dressings. J Plast Reconstr Aesthet Surg 2012;65:1377–83. 10.1016/j.bjps.2012.04.020. 22633389

[11] Senchenkov A , Knoetgen J, Chrouser KL, et al. Application of vacuum-assisted closure dressing in penile skin graft reconstruction. Urology 2006;67:416–9. 10.1016/j.urology.2005.08.037. 16461101

[12] Liguori G , Papa G, Boltri M, et al. Reconstruction of penile skin loss using a combined therapy of negative pressure wound therapy, dermal regeneration template, and split-thickness skin graft application. Int J Impot Res 2020;33:854–9. 10.1038/s41443-020-00343-1. 32801347

[13] Jeyaraman M , Muthu S, Sharma S, et al. Nanofat: a therapeutic paradigm in regenerative medicine. World J Stem Cells 2021;13:1733–46. 10.4252/wjsc.v13.i11.1733. 34909120 PMC8641019

